# Novel Glutamate–Putrescine Ligase Activity in *Haloferax mediterranei*: A New Function for *glnA-2* Gene

**DOI:** 10.3390/biom11081156

**Published:** 2021-08-04

**Authors:** Verónica Rodríguez-Herrero, Arnau Peris, Mónica Camacho, Vanesa Bautista, Julia Esclapez, María-José Bonete

**Affiliations:** 1 Agrochemistry and Biochemistry Department, Biochemistry and Molecular Biology Division, Faculty of Science, University of Alicante, 03080 Alicante, Spain; veronicarodriguezh13@gmail.com (V.R.-H.); camacho@ua.es (M.C.); vanesa.bautista@ua.es (V.B.); 2 Institute for Integrative Systems Biology, I2SysBio, Campus Burjassot, University of Valencia-CSIC, 46908 Valencia, Spain; arnauperis92@gmail.com

**Keywords:** glutamine synthetase, glutamate–putrescine ligase, nitrogen assimilation, putrescine, haloarchaea

## Abstract

The genome of the halophilic archaea *Haloferax mediterranei* contains three ORFs that show homology with glutamine synthetase (GS) (*glnA-1*, *glnA-2*, and *glnA-3*). Previous studies have focused on the role of GlnA-1, suggesting that proteins GlnA-2 and GlnA-3 could play a different role to that of GS. Glutamine synthetase (EC 6.3.1.2) belongs to the class of ligases, including 20 subclasses of other different enzymes, such as aspartate–ammonia ligase (EC 6.3.1.1), glutamate–ethylamine ligase (EC 6.3.1.6), and glutamate–putrescine ligase (EC 6.3.1.11). The reaction catalyzed by glutamate–putrescine ligase is comparable to the reaction catalyzed by glutamine synthetase (GS). Both enzymes can bind a glutamate molecule to an amino group: ammonium (GS) or putrescine (glutamate–putrescine ligase). In addition, they present the characteristic catalytic domain of GS, showing significant similarities in their structure. Although these proteins are annotated as GS, the bioinformatics and experimental results obtained in this work indicate that the GlnA-2 protein (HFX_1688) is a glutamate–putrescine ligase, involved in polyamine catabolism. The most significant results are those related to glutamate–putrescine ligase’s activity and the analysis of the transcriptional and translational expression of the *glnA-2* gene in the presence of different nitrogen sources. This work confirms a new metabolic pathway in the *Archaea* domain which extends the knowledge regarding the utilization of alternative nitrogen sources in this domain.

## 1. Introduction

Glutamine synthetase (GS; EC 6.3.1.2), which belongs to the class of ligases, can form carbon–nitrogen bonds using ATP. This class includes 20 different subclasses of enzymes, including GS, aspartate–ammonia ligase (EC 6.3.1.1), glutamate–ethylamine ligase (EC 6.3.1.6), and glutamate–putrescine ligase (EC 6.3.1.11). GS is an essential enzyme in nitrogen metabolism that catalyzes the synthesis of glutamine from glutamate and ammonium. Glutamine biosynthesis occurs through the biosynthetic reaction that first involves the formation of γ-glutamyl-phosphate from ATP and glutamate and, later, the release of phosphate, resulting in l-glutamine [1]. GS is a metalloenzyme dependent on ATP and divalent metal ions such as magnesium (Mg^2+^) or manganese (Mn^2+^), obtaining greater effectiveness via its catalytic activity in vitro in the presence of Mn^2+^ [2,3,4]. GS acts with the glutamate synthase enzyme (GOGAT; EC 1.4.7.1), catalyzing the reductive transfer of the amide group from l-glutamine to 2-oxoglutarate. This reaction, which is dependent on reducing power, generates two molecules of l-glutamate, one of which is recycled as a substrate for the GS reaction, while the other is exported or used to produce other amino acids [5]. GS genes show homology in different organisms, even in distant evolutionary species; hence, this enzyme has been considered an excellent molecular clock in gene evolution [6,7,8,9]. Different organisms present between one and four different GS, which encode as *glnA* genes; however, many of these genes are incorrectly annotated and the proteins are involved in other types of functions within nitrogen metabolism.

In recent years, *Haloferax mediterranei* has become one of the best-known halophilic microorganisms belonging to the *Archaea* domain and is considered to be a model organism for studying nitrogen metabolism [10,11,12,13,14,15,16,17,18,19,20], in addition to tolerance to environmental stress agents [21], thus making it a promising candidate for future biotechnological applications. In relation to nitrogen metabolism, *Hfx. mediterranei* assimilates ammonium through two different pathways depending on its availability in the cell. When ammonium is present at low concentrations, it is incorporated into carbon skeletons by GS-GOGAT. However, when ammonium is present at high concentrations, it is assimilated through the glutamate dehydrogenase enzyme (GDH; EC 1.4.1.2) [12,17]. It is known that the genome of *Hfx. mediterranei* encodes three *glnA* genes (*glnA-1*, *glnA-2,* and *glnA-3*), whose sequences show homology with the GS sequences of various organisms [14].

Previously, conditional mutants of the *Hfx. mediterranei glnA-1* gene (HM26-Δ*glnA-1*) were obtained in the presence of glutamine in culture media [20]. On the contrary, without the addition of glutamine, the mutants obtained were heterozygous, containing two versions of the gene (wild and deleted) in their genome. This fact highlights the necessity of the *glnA-1* gene in *Hfx*. *mediterranei.* HM26-Δ*glnA-1* transcriptome analysis revealed that genes *glnA-2* and *glnA-3* did not present the same expression profile, so the role of *glnA-2* and *glnA-3* remains unclear [20]. The protein encoded by the *glnA-1* gene has an identity of 51.9% and 49.1%, with the proteins encoded by the *glnA-2* and *glnA-3* genes, respectively. GlnA-2 and GlnA-3 proteins show an identity between them of 60.9%. The GlnA-1 protein from *Hfx. mediterranei* maintains partially or completely conserved the three consensus sequences characteristic of GS: PS00180 (glutamine synthetase signature 1), PS00181 (putative ATP binding region signature), and PS00182 (class I adenylation site). However, the GlnA-2 and GlnA-3 proteins only partially conserve one of them (PS00181) [14]. In addition, it was determined that the three *Hfx. mediterranei* GlnA proteins present the catalytic domain PF00120 (Gln-Synt_C) used to identify GS type I (GSI). This domain is found in GS proteins and another class of proteins related to polyamine metabolism. Universally, the GSI presents 18 conserved amino acids which are also used to identify these proteins. Amino acid sequence analysis of the three *Hfx*. *mediterranei* GlnA reveals that the sequence of the GlnA-1 protein presents the 18 conserved residues, in addition to the typical adenylylation residue (Y_385_) involved in its enzymatic activity regulation. However, eight of the key residues for glutamine biosynthesis are replaced by others in the GlnA-2 protein, and lack the adenylylation residue in both proteins.

Some species, such as *Streptomyces coelicolor*, *Myxococcus xanthus,* and *Pseudomonas* KIE171, present different proteins that show remarkable similarity with GS (between 30–50%) and contain specific catalytic domains, but do not show GS activity. These proteins can catalyze reactions in a comparable manner to the glutamine synthesis reaction but using different substrates to ammonium and/or glutamate. In *S. coelicolor* M145, it has been demonstrated that the GlnA3 protein (SCO6962), first annotated as GS, is involved in the catabolism of polyamines, and is expressed under ammonium limitation conditions and with low glucose concentration [22]. Another GS-like protein from *S. coelicolor* M145, GlnA4 (SCO1613), is involved in the metabolism of a new ethanolamine pathway, in which the GlnA4 protein acts as a γ-glutamyl–ethanolamide synthetase [23].

The polyamine catabolism has been studied mainly in Gram-negative bacteria. Different degradative pathways for polyamines in prokaryotes are known, including the γ-glutamylation pathway, the aminotransferase pathway, the spermine/spermidine acetyltransferase pathway, and the direct oxidation pathway [24,25,26,27,28,29,30,31,32]. In *Escherichia coli* and *Pseudomonas aeruginosa*, putrescine degradation is carried out by forming succinate using γ-aminobutyrate (GABA) as an intermediate metabolite through two pathways, the γ-glutamylation pathway and the aminotransferase pathway [29,30,31,32]. In addition, *P. aeruginosa* has an extended polyamine degradative pathway that involves seven γ-glutamyl-polyamine synthetases (PauA1-7), which are specific for the different polyamines, monoamines, or other substrates [31]. The distribution of polyamines among the different groups of *Archaea* is characteristic of each of them. Hyperthermophilic, acidophilic, and thermoacidophilic archaea contain a significant diversity of linear polyamines, whereas methanogenic archaea contain homospermidine, putrescine, and more commonly, spermidine [33,34,35]. Some of the first observations on polyamines related to archaea determined that halobacteria lacked polyamines [36,37].

This work shows that *Hfx*. *mediterranei* is able to grow in the presence of alternative nitrogen sources, such as putrescine, as the only source of nitrogen or carbon. In addition, this study provides new insight about the GlnA-2 role, which exhibits a novel glutamate–putrescine ligase activity instead of GS activity, and represents the first time that this activity has been detected in *Archaea* domain.

## 2. Materials and Methods

### 2.1. Bioinformatic Analysis

For the selection of the amino acid sequences, three independent alignments for each *Hfx. mediterranei* glutamine synthetase—GlnA-1 (HFX_0245), GlnA-2 (HFX_01688), and GlnA-3 (HFX_01686)—were carried out by BLASTP [38] against the NCBI database of “non-redundant protein sequences (nr)”. Sequences with a percent identity greater than 95% between them were eliminated. Phylogenetic inference was performed using the maximum likelihood method and the Le Gascuel model [39]. Finally, the phylogenetic tree with the highest logarithmic probability value was selected. A total of 500 re-samples were used by bootstrapping [40]. The initial tree for the heuristic search was automatically obtained using the Neighbor-Join (NJ) algorithm from a matrix of pairwise distances estimated using the JTT model and, subsequently, by selecting the topology corresponding to the highest log-likelihood value.

For the identification of conserved residues and domains present in the GlnA proteins from *Hfx. mediterranei,* the alignment against GS model protein of *Salmonella typhimurium* was carried out. Furthermore, an in silico analysis focused on the identification of conserved domains in the GS was performed using different tools: HMMER [41] and Prosite Scan [42].

### 2.2. Strains and Culture Conditions

*Hfx. mediterranei* R4 (ATCC 33500T) was grown at 42 °C with aeration at 250 rpm. The culture media contained 25% (*w*/*v*) seawater [43] with different nitrogen sources and, in the absence of a nitrogen source (Table 1), was supplemented with 5 g/l glucose, 0.0005 g/L FeCl_3_, and 0.5 g/L KH_2_PO_4_. The pH value was adjusted to 7.3 with NaOH. For the preparation of the medium in the absence of nitrogen, cells from a culture with nitrogen source were harvested by centrifugation at 13,000 rpm for 30 min, washed with 25% seawater, and then transferred to a medium without a nitrogen source to induce the nitrogen starvation. Cells were subjected to nitrogen starvation for 120 h.

*Hfx*. *mediterranei* HM26 (R4-Δ*pyrE2*) was grown in the same conditions. In addition, the culture media to grow the HM26 strain were supplemented with 50 μg/mL uracil [15,20].

*E. coli* strains DH5α for cloning and JM110 for efficient transformation of *Hfx*. *mediterranei* were grown overnight in Luria—Bertani medium with ampicillin (100 mg/mL) at 37 °C.

### 2.3. Identification and Expression of glnA-1 and glnA-2 Promoter Regions

The identification of the possible TATA boxes, BRE sequences, and transcription initiation sites for each of the genes was carried out using a combination of the different bioinformatic tools for bacteria and eukaryotes. In addition, a manual search was performed based on consensus sequences for TATA boxes and halophilic BRE sequences [44,45,46,47]. From *Hfx. mediterranei* R4 genomic DNA, the promoter regions identified of the *glnA-1* and *glnA-2* genes were amplified with the specific primers for each of these areas, including the cut-off points of the restriction enzymes *Hind*III and *Nco*I (Table S1). The cloning vector pGEM-T Easy (Promega, Barcelona, Spain) and the halophilic vector pVA513 (kindly provided by Dr Mike Dyall-Smith (University of Melbourne, Australia)) were used, in addition to the chemically competent cells *E. coli* DH5α and JM110 (Promega, Barcelona, Spain). The halophilic vector pVA513 has origins of replication for both *E. coli* (p*BR322*) and *Haloferax sp.* (p*HK2*), an ampicillin resistance gene to work in *E. coli* (Amp^R^), a novobiocin resistance gene to work in *Haloferax sp.* (Nov^R^), and *Hind*III and *Nco*I restriction enzymes cleavage targets followed by the *Haloferax lucentense* β-galactosidase gene. The *Hfx. mediterranei* transformants with the constructions pVA513-p-*glnA-1* and pVA513-p-*glnA-2* were grown in Hm-CM, in Hm-DM with 40 mM ammonium or with 40 mM nitrate as the nitrogen source and, in Hm-NS. All media were supplemented with 0.3 µg/mL novobiocin. The cultures were carried out in triplicate, and growth was monitored by measuring the OD_600nm_ throughout the entire growth period. The characterization of the promoters was carried out by measuring the β-galactosidase activity and the cell-free extracts were processed as described in previous work [48,49]. All measurements were made in triplicate. One unit of β-galactosidase activity was defined as the amount of enzyme that catalyzes the hydrolysis of 1 μmol of ONPG min^−1^.

### 2.4. Gene Expression Analysis by Reverse Transcription PCR

RNA was isolated from *Hfx*. *mediterranei* R4 strain in Hm-CM cultures and in Hm-DM in the presence of two different nitrogen sources, 40 mM nitrate or 40 mM ammonium, in the middle of the exponential phase and the stationary phase. RNA was isolated after nitrogen starvation for 48, 96, and 120 h from the *Hfx. mediterranei* R4 strain in Hm-NS cultures. Total RNA isolation, quality, and quantity were analyzed as described in previous work [50]. Four independent biological replicates of each condition were performed. For cDNA synthesis, an RNA sample (0.5–0.6 μg) and M-MuLV Reverse Transcriptase (Thermo Scientific, Waltham, MA, USA) were used. Negative controls were performed without enzyme or RNA. The RT-PCR protocol was performed according to the manufacturer’s instructions. The oligonucleotides used to perform the RT-PCR were designed based on the *glnA-1* and *glnA-2* genes (Table S1).

### 2.5. Proteins Expression Analysis by Western Blotting and Molecular Weight Determination

Western blotting was performed as described in the Western blotting principles and methods manual (GE Healthcare, Chicago, IL, USA) using 30 µg of protein extracts, obtained from *Hfx. mediterranei* R4 cultures in Hm-DM in the presence of different nitrogen sources (40 mM nitrate, 40 mM ammonium, or 5–80 mM putrescine). Protein extracts obtained from *Hfx*. *mediterranei* R4 cultures in the absence of a nitrogen source (Hm-NS) for 48, 96, and 120 h were also used.

Harvested cells were resuspended to 40% (*w*/*v*) in 20 mM Tris–HCl, 2 M NaCl, and 10 mM MgCl_2_ buffer pH 7.5. As a primary antibody (polyclonal rabbit anti-GlnA antibody), two synthetic peptides (GenScript, NJ, USA) were used at a concentration of 0.5 μg/mL, and a secondary antibody was labeled with peroxidase 1:50,000 (Thermo Scientific, Waltham, MA, USA), which uses luminol as a chemiluminescent substrate (GE Healthcare, Chicago, IL, USA). Recombinant GlnA proteins obtained by heterologous expression in *E. coli* were used as the positive control [14,51]. Moreover, recombinant GlnA-2 quaternary structure verification was analyzed by gel filtration chromatography using a HiPrep 16/60 Sephacryl S-300 High-Resolution column (GE Healthcare, Chicago, IL, USA) previously equilibrated with 20 mM Tris-HCl pH 7.0, 2 M NaCl. Standard proteins for gel filtration chromatography from 12.5 to 678 kDa were used as markers to estimate the protein molecular mass. Protein was eluted in the presence of salt, at a flow rate of 0.5 mL/min in 20 mM Tris–HCl, 2 M NaCl and 10 mM MgCl_2_ buffer pH 7.5. All of the purification steps were carried out at room temperature.

### 2.6. Assays for GlnA Protein: Enzymatic Activity

**Scheme 1:**
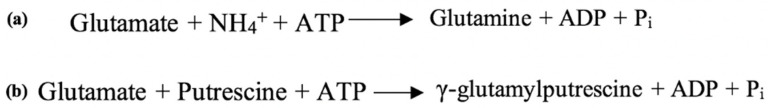
Schematic reaction of enzyme activities. (**a**) GS biosynthetic reaction. (**b**) Glutamate–putrescine ligase reaction.

GS and glutamate–putrescine ligase activities were measured using the method described by Shapiro and Stadtman [52]. In both reactions, inorganic phosphate is produced from the consumption of ATP. The schematic GS biosynthetic reaction to produce glutamine is shown in Scheme 1a, and the glutamate–putrescine ligase reaction to produce γ-glutamylputrescine is shown in Scheme 1b.

The reaction mixtures were incubated at 42 °C for 15 min. The final product was detected at 660 nm. This assay was performed with both recombinant GlnA proteins and cell extracts from *Hfx. mediterranei* R4 growth in the presence of different nitrogen sources. All tests were carried out in triplicate. Negative control assays were also performed in parallel with the absence of the enzyme or the absence of ATP. One unit of GS activity was defined as the amount of protein that produces 1 μmol of phosphate/min.

### 2.7. Construction and Characterization of the HM26-∆glnA-2 Mutants

In-frame deletion mutants of the *glnA-2* gene (HM26-Δ*glnA-2*) were obtained from the parental strain *Hfx. mediterranei* HM26 (R4-Δ*pyrE2*) using the pop-in/pop-out method as described previously for *Hfx*. *mediterranei* [15,20,53]. For the characterization of the HM26-Δ*glnA-2* strain, the growth and stability of the mutant in Hm-CM and Hm-DM in the presence of different nitrogen sources, i.e., 5–80 mM nitrate, 5–80 mM ammonium, or 5–80 mM putrescine, were studied. The statistical analysis of the growth parameters of the HM26-Δ*glnA-2* strain was carried out compared to the parental strain *Hfx. mediterranei* HM26 (R4-Δ*pyrE2*) obtained in previous work [15] under the same conditions. Three biological replicates were performed for each strain and culture medium. The stability of *glnA-2* deletion during growth on different culture media was determined by PCR screening, Southern blotting, and Western blotting.

## 3. Results

### 3.1. Bioinformatic Analysis of the GS Proteins

**Figure 1:**
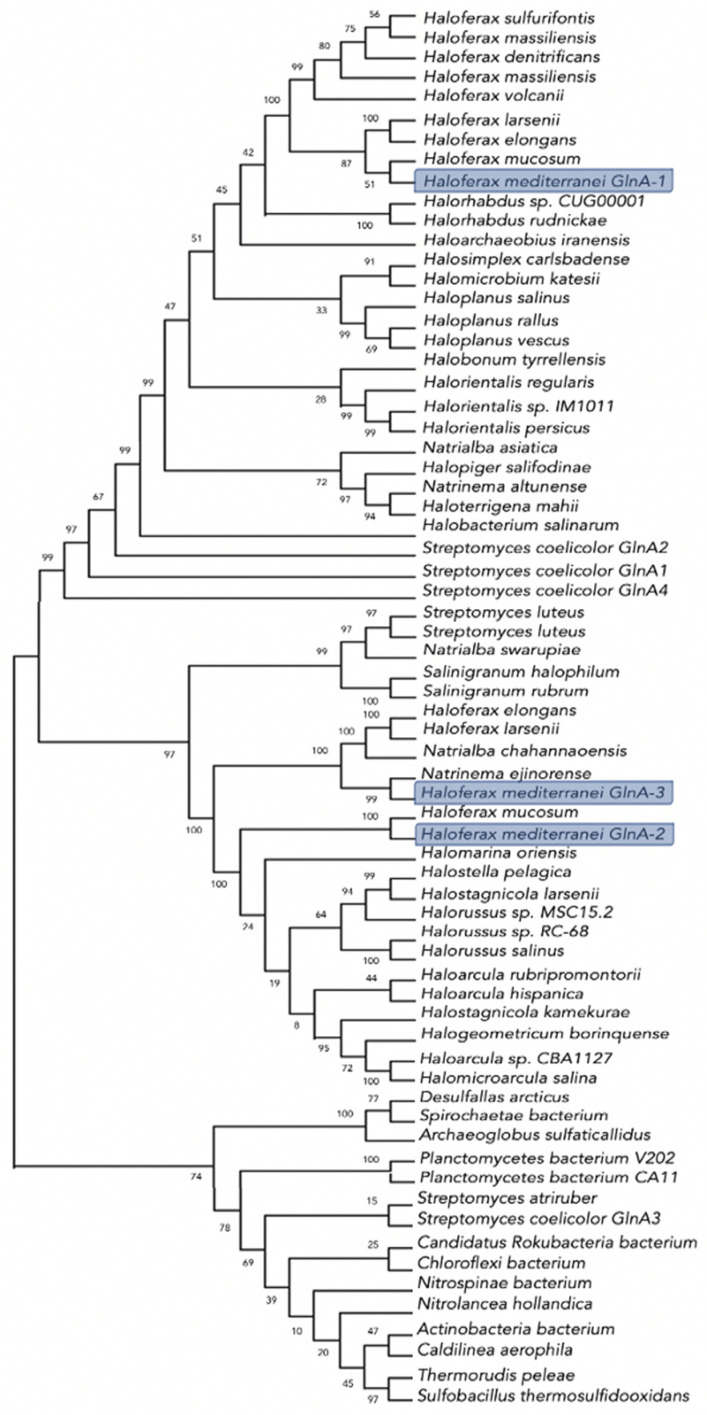
Phylogenetic tree of the GlnA proteins based on the amino acid sequence. The three *Hfx*. *mediterranei* GlnA proteins are highlighted in blue.

The phylogenetic analysis revealed that at the origin of the obtained phylogenetic tree (Figure 1), there is a bifurcation differentiating two central nodes. In the first node are all the GlnAs from species belonging to the *Archaea* domain, with the exception of two bacterial species (*S. coelicolor* and *Streptomyces luteus*). Two other well-differentiated secondary nodes emerge from the first node. Most of the proteins found in the different branches of the first secondary node are species from the *Haloferacaceae* family, which includes the GlnA-1 protein from *Hfx*. *mediterranei*. Most of the proteins found in the other secondary node are species from the *Halobacteriaceae* family and other representatives of the *Haloferacaceae*, *Natrialbaceae,* and *Halorubraceae* families (according to abundance order). This node contains the GlnA-2 and GlnA-3 proteins from *Hfx. mediterranei*, which are phylogenetically closer to each other than GlnA-1. Several GlnA proteins, which are present in the same organism, are located at different nodes, as occurs with the GlnA proteins of *Hfx. mediterranei,* where GlnA-1 is located at a different node than GlnA-2 and GlnA-3. These results suggest an ancient duplication of *Hfx. mediterranei* GlnA proteins and subsequent diversification of the paralogs on the two main branches of the tree.

**Figure 2:**
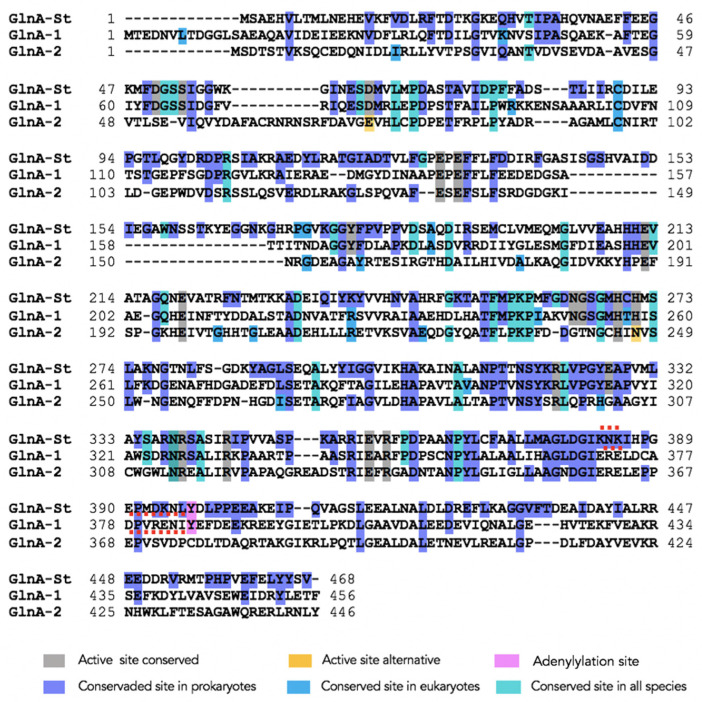
Identification of the conserved residues of *Hfx*. *mediterranei* GlnA-1 and GlnA-2 proteins. The adenylylation box is shown marked with a dashed red line. GlnA-St corresponds to the GS protein sequence from *S. typhimurium*.

To elucidate whether the *glnA* genes encode functional GS, the amino acid sequences were aligned against the sequence of the *S. typhimurium* GlnA, determining the presence or absence of the amino acid residues characteristic of the active site of GS. The sequence of *S. typhimurium* GlnA (P0A1P6) was established as a reference model, given that its structure is determined by X-ray crystallography. It shows 37.6% and 28.0% identity with GlnA-1 and GlnA-2, respectively. The alignment results show that GlnA-1 contains 18 conserved residues, whereas GlnA-2 contains 10 of these 18 conserved residues (Figure 2).

There are 35 and 187 conserved amino acids in the GS of most species and prokaryotes, respectively. GlnA-1 contains 28 (80.0%) conserved residues and GlnA-2 contains 21 (60.0%), whereas 84 (44.9%) and 48 (25.7%) of the residues present in prokaryotes are conserved in GlnA-1 and GlnA-2, respectively. The residue substitutions could be due to the mechanisms of adaptation and divergence processes. The adenylylation box is composed of eight universally conserved residues (KNKPDKLY). GlnA-1 presents five residues of the adenylylation box, including the tyrosine residue (Y_397_), whereas the *Hfx. mediterranei* GlnA-2 protein only has three residues, lacking the adenylylation residue (Y_397_), which is replaced by the C_375_ residue.

The conserved residues and their relative positions (numbering refers to *S. typhimurium* GlnA) present in each GlnA protein of *Hfx*. *mediterranei* are summarized in Table 2, confirming that GlnA-2 does not present essential residues for glutamine synthesis (E_327_ and D_50_). Furthermore, the Y_179_ residue essential for binding to ammonium is replaced by alanine, providing significantly more space for binding with a bulky substrate. These results suggest that GlnA-1 protein from *Hfx. mediterranei* corresponds to functional GS as previously described [20], whereas GlnA-2 may be involved in other functions. By comparison, *Hfx*. *mediterranei* GlnA-1 and GlnA-2 protein sequence analysis by InterProScan1 predicted a beta-C-terminal catalytic domain corresponding to the characteristic GS enzymatic domain (IPR008146). This domain is present in many proteins of the GS family.

### 3.2. Identification and Expression of glnA-1 and glnA-2 Promoter Regions

**Scheme 2:**
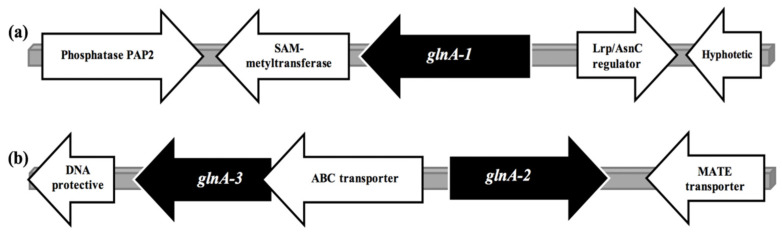
Genomic organization of *Hfx*. *mediterranei glnA* genes. (**a**) Genomic organization of the *glnA-1* gene. (**b**) Genomic organization of the *glnA-2* gene.

The genomic organization analysis determined that the *glnA-1* gene (Scheme 2a) appears in the genome located downstream of the Lrp/AsnC transcriptional regulator [49] and upstream of SAM-methyltransferase genes. The *glnA-2* gene (Scheme 2b) is located between two transporter genes (downstream of the MATE transporter and upstream of the ABC transporter family), and close to the *glnA-3* gene; both are distant from the *glnA-1* gene.

The promoter regions of the *glnA-1* and *glnA-2* genes were identified by bioinformatic analysis. The selected regions contain the possible TATA box and the BRE sequence, in addition to the transcriptional start site and the start of the gene (Figure S1). These promoter regions were cloned in the halophilic vector pVA513. The *Hfx. mediterranei* transformants with the pVA513-p-*glnA-1* (Figure S2) and pVA513-p-*glnA-2* (Figure S3) constructions were characterized. The growth of these transformants was similar to those described previously. The characterization of the promoter region of the *glnA* genes was carried out by measuring specific β-galactosidase activity using different cultures of *Hfx mediterranei* growth under several nitrogen sources. β-galactosidase activity was detected in all media analyzed at the different growth times (Figures S2 and S3).

In Hm-CM and Hm-DM with 40 mM ammonia or nitrate, the transformants with the pVA513-p-*glnA-1* construction showed the maximum specific β-galactosidase activity in the middle of the exponential phase. From that point, the activity of β-galactosidase decreased, although it was detected until reaching the stationary phase. In the absence of nitrogen (Hm-NS), as expected, the transformants with the pVA513-p-*glnA-1* construction showed higher β-galactosidase activities than in the presence of ammonium as a nitrogen source. Under nitrogen starvation conditions, the β-galactosidase activity was maintained for up to 120 h, obtaining the best values of specific activity at 24 h of nitrogen deficiency (Table 3, Figure S2).

The transformants with the pVA513-p-*glnA-2* construction (Figure S3) showed β-galactosidase activity values that were very similar in all the conditions tested independently of the nitrogen source. Maximum activity values were detected in the middle of the exponential growth phase in all the conditions. Under nitrogen starvation conditions, β-galactosidase activity values did not show changes. Regardless of the number of hours of starvation of the nitrogen source, these values were between 2.6 ± 0.3 and 2.9 ± 0.2 U/mg. Furthermore, in this condition, the β-galactosidase activity values were maintained up to 120 h. These results suggest that the *glnA-1* expression appears to increase when the ammonium concentration decreases, whereas the *glnA-2* expression appears to be similar in all of the conditions analyzed.

The β-galactosidase activity was also analyzed in the presence of 40 mM putrescine (Figure S4). Surprisingly, *Hfx. mediterranei* can grow in Hm-DM with 40 mM putrescine as a nitrogen source. Under this culture condition, the transformants with the pVA513-p-*glnA-1* and pVA513-p-*glnA-2* constructions showed β-galactosidase activity at different growth phases. The maximum activity values were detected in the middle of the exponential growth phase in this condition (Table 3).

### 3.3. Gene Expression Analysis by Reverse Transcriptase PCR

The expression of *glnA-1* and *glnA-2* genes from *Hfx. mediterranei* was analyzed by RT-PCR using RNA isolated from cultures grown in the presence of different nitrogen sources.

**Figure 3:**
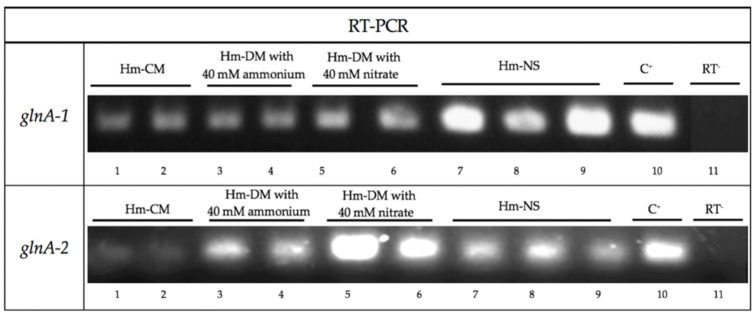
Analysis of the *Hfx. mediterranei glnA* genes expression by RT-PCR depending on nitrogen source. Lines 1-3-5: mid-exponential phase. Lines 2-4-6: stationary phase. Line 7: 48 h nitrogen starvation. Lines 8: 96 h nitrogen starvation. Line 9: 120 h nitrogen starvation. Line 10: Positive control, DNA from *Hfx*. *mediterranei* strain R4. Line 11: Negative control, without reverse transcriptase.

The results obtained in this analysis show that the *glnA-1* and *glnA-2* genes are expressed in Hm-CM and in Hm-DM with different nitrogen sources (40 mM ammonium or 40 mM nitrate) at different growth phases (in the middle of the exponential phase and the stationary phase). As expected, under nitrogen starvation, *glnA-1* and *glnA-2* genes are expressed independently of the nitrogen deficiency time (48, 96, and 120 h). The results showed that both genes are constitutively expressed in all conditions tested (Figure 3) and are in agreement with those obtained in the characterization of the promoter regions, in which β-galactosidase activity was obtained in all of the analyzed media at different growth phases.

### 3.4. Proteins Expression Analysis by Western blotting and Molecular Weight Determination

Protein expression analysis was performed using *Hfx. mediterranei* R4 protein extracts from cultures grown under different conditions to analyze the GlnA-1 and GlnA-2 translational expression profiles depending on nitrogen source.

**Figure 4:**
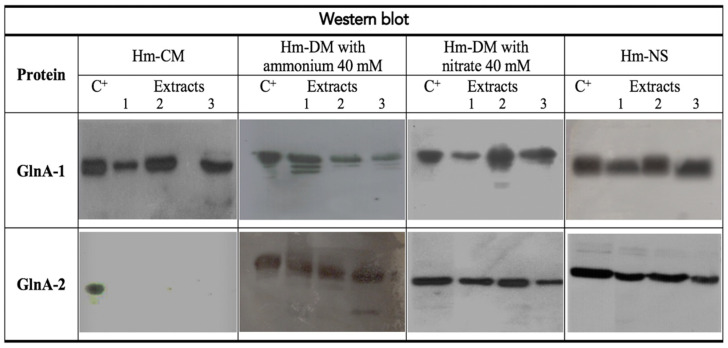
*Hfx. mediterranei* GlnA proteins expression depending on nitrogen source analyzed by Western blotting. The positive control (C^+^) corresponds to the recombinant protein of GlnA-1 and GlnA-2 proteins. Line 1: initial exponential phase. Line 2: mid-exponential phase. Line 3: stationary phase. In Hm-NS, the samples correspond to 1:48 h nitrogen starvation. 2:96 h nitrogen starvation. 3:120 h nitrogen starvation.

The Western blotting results show that GlnA-1 is expressed in Hm-CM independently of the growth phase. In Hm-DM, with 40 mM ammonium or 40 mM nitrate, the GlnA-1 protein also showed expression at the same growth phase (at initial exponential phase, at middle exponential phase, and in stationary phase). The GlnA-1 protein expression was detected in Hm-NS at 48, 96, and 120 h of nitrogen starvation (Figure 4).

The GlnA-2 protein expression was detected in all conditions (Hm-DM with 40 mM ammonium or nitrate and Hm-NS) at different growth phases, except in Hm-CM, in which the only expression was obtained in the positive control (Figure 4). This result suggests that GlnA-2 expression could be regulated at the post-transcriptional level, because GlnA-2 is expressed at the transcriptional level in Hm-CM.

Curiously, *Hfx*. *mediterranei* grew in Hm-DM with putrescine as the only source of nitrogen or carbon. These results indicate that *Hfx. mediterranei* can grow with polyamines. However, the maximum OD_600nm_ values reached were lower than those obtained in the presence of other nitrogen or carbon sources (Figure S5).

**Figure 5:**
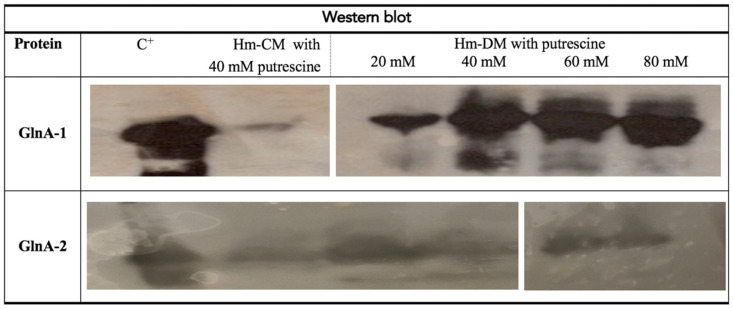
Expression of the GlnA proteins in the presence of different concentrations of putrescine by Western blotting. The positive control (C^+^) corresponds to the recombinant protein of GlnA-1 and GlnA-2 proteins.

Western blotting analysis carried out using *Hfx. mediterranei* protein extracts, in the presence of different concentrations of putrescine (20–80 mM) as a nitrogen source, indicate that the GlnA-1 and GlnA-2 proteins also showed expression in the middle of the exponential growth phase (Figure 5). In Hm-CM, in the presence of 40 mM putrescine in the same growth phase, the expression of the GlnA-1 and GlnA-2 proteins could also be detected. Consequently, these results are an excellent starting point to determine the presence of a new pathway for putrescine degradation in Haloarchaea.

In parallel, the molecular weight of recombinant GlnA-2 protein was calculated using size exclusion chromatography, being 667 kDa (Figure S6). As the molecular weight of GlnA-2 monomer was found to be 55 kDa by SDS-PAGE analysis, it suggests that this protein forms a dodecamer, as does GS. Although the GlnA-2 molecular weight presents a high degree of similarity with that of GS protein, it may not be a GS protein. Similar results have been described in *E. coli,* in which the GS protein and the glutamate–putrescine ligase (PuuA) share structural similarities [54].

### 3.5. GS Activity and Glutamate–Putrescine Ligase Activity Assays

**Figure 6:**
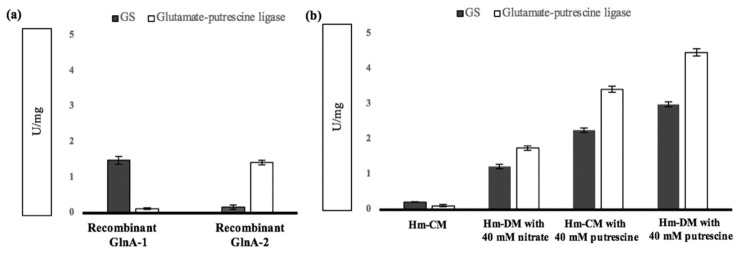
Assays for GlnA specific activity. (**a**) GS activity and glutamate–putrescine ligase activity in the recombinant proteins. (**b**) GS activity and glutamate–putrescine ligase activity depending on the nitrogen source.

As expected, GlnA-1 recombinant protein shows GS activity (1.48 ± 0.11 U/mg) (Figure 6a). In Hm-CM, the GS activity obtained (0.22 ± 0.01 U/mg) is lower than those obtained in other conditions, because the GS-GOGAT pathway is more active under nitrogen-deficient conditions. In Hm-DM with 40 mM nitrate or 40 mM putrescine, and in Hm-CM in the presence of putrescine, higher levels of GS activity were obtained (1.23 ± 0.06 U/mg, 2.99 ± 0.07 U/mg and 2.26 ± 0.06 U/mg, respectively) (Figure 6b); however, surprisingly, GlnA-2 recombinant protein shows only glutamate–putrescine ligase activity (Figure 6a). In Hm-CM, no glutamate–putrescine ligase activity was obtained (0.11 ± 0.03 U/mg) because GlnA-2 protein is not expressed in this condition; whereas, in Hm-DM with 40 mM nitrate, glutamate–putrescine ligase activity was clearly detected (1.75 ± 0.07 U/mg). Independently of the medium composition, the highest values of glutamate–putrescine ligase activities were obtained in the presence of 40 mM putrescine (3.42 ± 0.09 U/mg in Hm-CM with putrescine or 4.47 ± 0.11 U/mg in Hm-DM with putrescine) (Figure 6b).

At other concentrations of putrescine used (5–80 mM), both the GS activity and the glutamate–putrescine ligase activity were also obtained (data not shown). These results suggest that GlnA-2 protein from *Hfx*. *mediterranei* may be responsible for glutamate–putrescine ligase activity and is the first time that this activity has been evidenced in *Archaea*. However, to date, the role that this activity plays in the archaea is still unknown because glutamate–putrescine ligase activity has not been described in *Hfx. mediterranei* or in any other Haloarchaea.

### 3.6. Construction and Characterization of the HM26-∆glnA-2 Mutants

To determine the GlnA-2 protein role in *Hfx. mediterranei* metabolism, deletion mutants of the *glnA-2* gene were generated and characterized depending on nitrogen source. The Southern blot results confirm that the gene encoding GlnA-2 was deleted (Figure S7). The fact that the knockout mutants of the *glnA-2* gene were obtained without adding glutamine to the culture medium, in contrast to the *glnA-1* gene deletion mutants [20], indicates that the *glnA-2* gene is not an essential gene for glutamine synthesis in *Hfx. mediterranei*. The deletion mutants HM26-Δ*glnA-2* were characterized in Hm-DM with different ammonium, nitrate, or putrescine concentrations as the sole nitrogen source (data not shown). Statistical analysis revealed that the HM26-Δ*glnA-2* strain showed no significant differences in growth rate in Hm-CM. On the contrary, in Hm-CM with 40 mM putrescine, the differences were significant (Figure S8a). Moreover, the HM26-Δ*glnA-2* strain only showed significant differences in growth rate at high ammonium (20–80 mM) (Figure S8b), and nitrate concentrations (40–80 mM) (Figure S8c), whereas in the presence of putrescine as a nitrogen source, significant differences were observed at all concentrations tested (5–80 mM) (Figure S8d).

To prove whether deletion of the *glnA-2* gene could affect enzyme activity, an activity analysis was performed using extracts obtained from HM26-Δ*glnA-2* strain cultures in different nitrogen sources (Hm-CM, Hm-CM with 40 mM putrescine, Hm-DM with 40 mM nitrate, or putrescine). Both GS biosynthetic activity and glutamate–putrescine ligase activity were measured to carry out these analyses.

As expected, in Hm-CM, similar GS activity was obtained in both the parental *Hfx*. *mediterranei* strain (HM26) and the mutant *Hfx*. *mediterranei* strain (HM26-Δ*glnA-2*). The same results were also obtained with the glutamate–putrescine ligase activity.

In nitrate-defined or putrescine-defined medium, GS activity was obtained (1.25 ± 0.01 U/mg and 2.23 ± 0.07 U/mg respectively) in the parental strain (HM26), indicating that this activity is due to GlnA-1. However, although GS activity was also detected in the mutant strain (HM26-Δ*glnA-2*), the values showed a remarkable decrease of 30–40%. In the same conditions, glutamate–putrescine ligase activity was obtained in the parental strain (1.75 ± 0.07 U/mg with nitrate or 3.47 ± 0.11 U/mg with putrescine).

**Figure 7:**
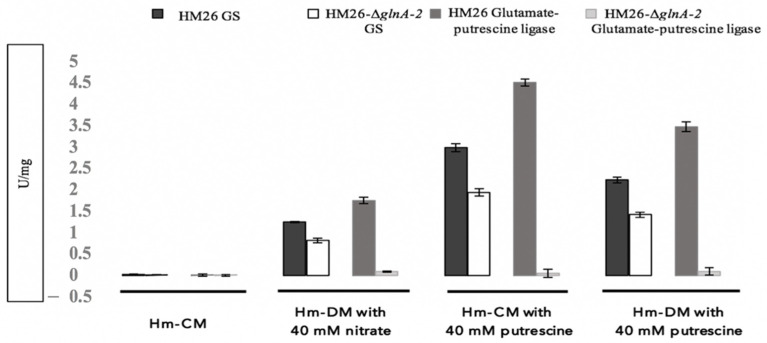
Comparison of GS and glutamate–putrescine ligase activities between *Hfx. mediterranei* parental strain (HM26) and mutant strain (HM26-Δ*glnA-2*), according to the nitrogen source.

Surprisingly, no glutamate–putrescine ligase activity was obtained in the HM26-Δ*glnA-2* mutant in these conditions. The same GS and glutamate–putrescine ligase activities results were obtained in Hm-CM with 40 mM putrescine, which did not show HM26-Δ*glnA-2* mutant strain glutamate–putrescine ligase activity. These results confirm that GlnA-2 exhibits putrescine activity rather than GS activity and, therefore, is a glutamate–putrescine ligase protein that is erroneously annotated as a GS protein (Figure 7).

## 4. Discussion

The *Hfx*. *mediterranei* genome has three genes annotated as GS (*glnA-1*, *glnA-2,* and *glnA-3*). Recently, it has been determined that the *glnA-1* gene is essential for its growth, whereas proteins GlnA-2 and GlnA-3 could play a different role [20]. In particular, *Hfx*. *mediterranei* GlnA-2 presents substitutions in crucial amino acids for glutamine synthesis and the lack of the typical adenylylation residue. Consequently, it may be possible that it plays a catalytic role in other types of reactions. Similar substitutions have been described in other proteins annotated as GS [55,56]. Therefore, this protein would not be subject to control by covalent modification mediated by adenylyltransferases, as also occurs in GlnA4 from *Myxococcus xanthus* and GlnA2, GlnA3, and GlnA4 from *S. coelicolor* [22,55,56]. Other examples of GS-like proteins have been described in *Pseudomonas* sp. KIE171, in which the IpuC protein annotated as GS-like, is involved in the degradation of isopropylamine [57]; in *E. coli* K-12, the *puuA* gene was initially annotated as GS, subsequently demonstrating that this gene encodes a glutamate–putrescine ligase [54]; and in *S. coelicolor* M145, the GlnA3 protein is involved in the degradation of polyamines encoding a γ-glutamylpolyamine synthetase [22], and the GlnA4 protein in the degradation of ethanolamine [23].

Based on β-galactosidase activity results, the expression of the *glnA-1* gene is indirectly related to the ammonium concentration, as occurs in *S. coelicolor* [58,59,60]. Furthermore, these results are in agreement with previous studies in which RT-PCR, qPCR, and microarray demonstrated that the transcription of the GS/GOGAT pathway genes and other genes involved in the nitrate assimilation pathway increased in nitrogen limiting conditions [13,16,17,61]. By comparison, these results indicate that the *glnA-1* gene in *Hfx. mediterranei* is constitutively expressed, showing basal β-galactosidase activity. Opposite results have been reported for the *Hfx*. *mediterranei nasA* promoter, which presents a maximum activity at the beginning of the exponential growth phase, and shows a remarkable decrease in activity during the exponential phase in Hm-DM with 40 mM nitrate. Very low levels of β-galactosidase activity under the p-*nas* promoter control were detected in Hm-DM with 40 mM ammonia; values were close to zero throughout, indicating that the promoter was inactive under these conditions and only presented a very low basal activity [48]. The expression of *glnA-2* appears not to depend on nitrogen limitation, unlike that of the *glnA-1* gene.

At the translational level, the GlnA-1 protein shows expression in all nitrogen sources analyzed. By comparison, although the *glnA-2* gene shows expression in all nitrogen sources analyzed, the GlnA-2 protein does not show expression at the translational level in Hm-CM. This could be because the *glnA-2* gene is controlled by some type of regulation at the transcriptional level. At present, sRNAs are known to play an essential role in the post-transcriptional regulation of many processes in the three domains of life [62,63,64]. Recently sRNAs (sRNA274 and sRNA310) were identified in *Hfx. mediterranei* whose expression patterns change according to the nitrogen source [18]. This study shows that the *glnA-2* gene is one of the potential target genes of these sRNAs, and it is possible that the expression of GlnA-2 would be regulated by this mechanism [19].

To elucidate the role of GlnA-2 in *Hfx. mediterranei* primary metabolism, its growth and expression were analyzed in the presence of other nitrogen sources, specifically in the presence of putrescine. This work shows that *Hfx. mediterranei* can grow in the presence of polyamine putrescine as the sole nitrogen or the sole carbon source. However, its growth is less than that using other nitrogen sources, such as ammonium or nitrate, or another carbon source, such as glucose. In addition, the β-galactosidase assay and Western blotting showed that GlnA-1 and GlnA-2 are expressed in Hm-DM in the presence of putrescine (20–80 mM). To test whether GlnA-2 can metabolize putrescine, GS activity and glutamate–putrescine ligase activity were assayed from recombinant GlnA-1 and GlnA-2 proteins. Results showed that only the GlnA-1 protein presents GS activity, whereas only the GlnA-2 protein presents glutamate–putrescine activity. Furthermore, in the mutant strain HM26-Δ*glnA-2*, the GS activity decreased approximately 30–40%, whereas the glutamate–putrescine ligase activity was almost undetectable. Higher activity values were obtained under nitrogen-limiting conditions for GS and glutamate–putrescine activities. At high concentrations of ammonium or nitrate, the growth of the mutant strain HM26-Δ*glnA-2* showed significant differences concerning the growth of the parental strain HM26. This may be because the products of the pathway in which the GlnA-2 protein is involved may serve as a substrate of the pathway in which the GlnA-1 protein is involved, or some of its products may share both pathways (polyamine degradation and ammonium assimilation). However, in Hm-CM, the growth of both strains was similar because, in this condition, the GlnA-2 protein is not expressed, because it is absent in the mutant strain, it has no effect. The results of this work, both bioinformatic and experimental, show that the *glnA-2* gene—previously annotated as a GS—encodes a glutamate–putrescine ligase protein.

**Figure 8:**
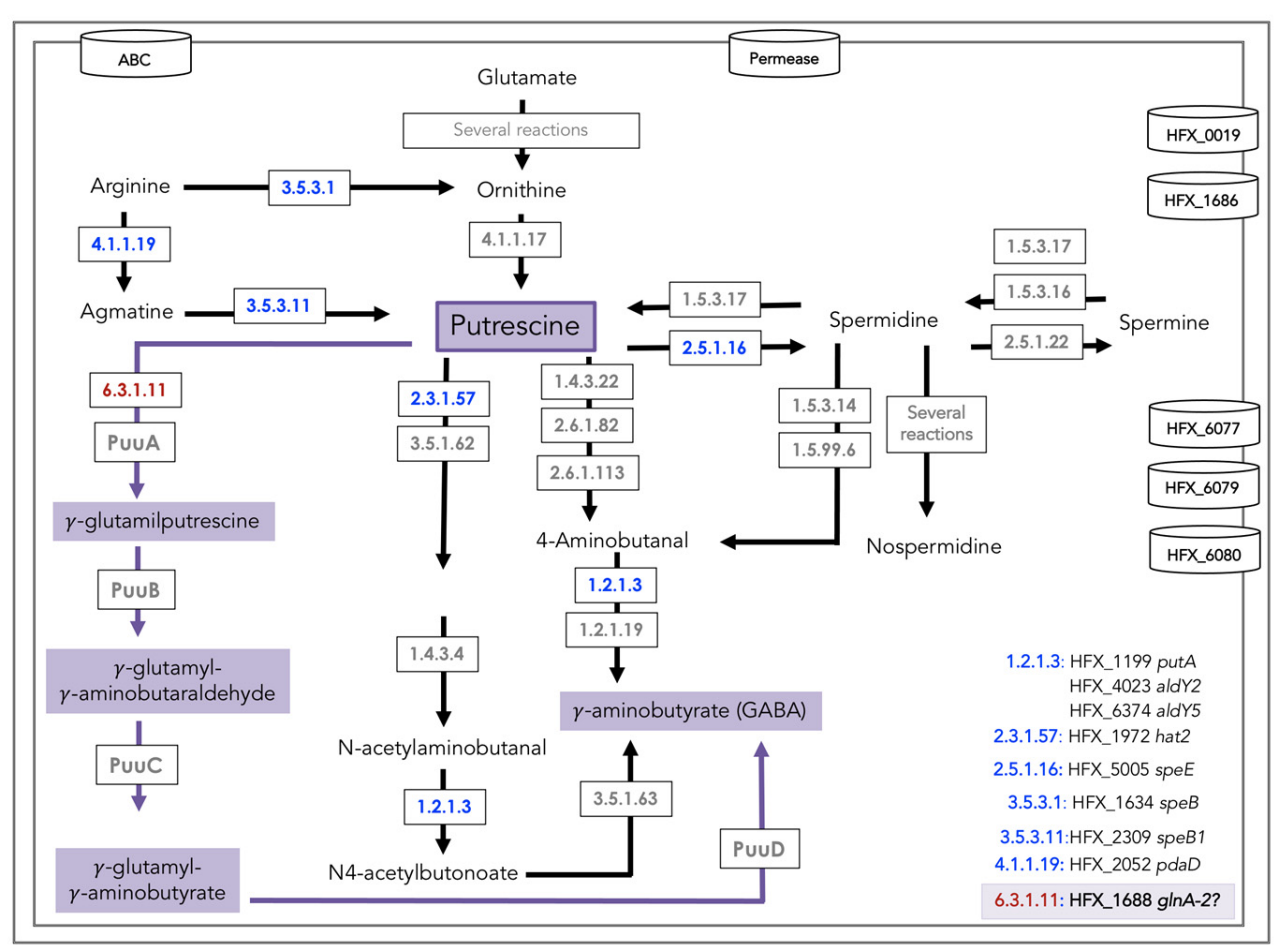
Representation of the possible metabolic pathways of putrescine degradation and other polyamines in *Hfx. mediterranei* R4. Image based on the metabolic maps included in the KEGG database (organism code: hme). In blue, the EC numbers of each of the enzymes whose coding genes are present in *Hfx. mediterranei* are indicated. In grey, the enzymes that catalyze these reactions whose genes are not present in the genome of this halophilic archaea. In red, the two genes that could encode glutamate–putrescine ligase. The white cylinder represents the different amino acid and polyamine transporters.

*Hfx. mediterranei* may present a putrescine degradation reaction similar to that of *E. coli*, in which the first step of glutamination would be catalyzed by GlnA-2 (Figure 8). The first step of the γ-glutamylation pathway, or Puu pathway in *E. coli,* consists of the glutamylation of putrescine to γ-glutamylputrescine catalyzed by γ-glutamylputrescine synthetase or glutamate–putrescine ligase (PuuA) (EC 6.3.1.11). The γ-glutamylputrescine is completely oxidized to γ-glutamylaminobutyrate by γ-glutamylputrescine oxidase (PuuB) and γ-butyraldehyde dehydrogenase (PuuC). The γ-glutamylaminobutyrate is then hydrolyzed to glutamate and γ-aminobutyrate (γ-GABA) by γ-GABA hydrolase (PuuD) [30,54,65]. However, the aminotransferase pathway requires, first, the transamination of putrescine with 2-oxoglutarate to generate glutamate and γ-aminobutyraldehyde by putrescine aminotransferase (PatA) and, second, the oxidation of this γ-aminobutyraldehyde to γ-aminobutyrate by γ-aminobutyraldehyde dehydrogenase (PatD/PuuC). In addition, different genes related to polyamine metabolism in this bacterium have been identified in the *Hfx*. *mediterranei* genome, such as *putA*, *aldY2*, *aldY5*, *hat2*, *speE*, *speB1*, and *pdaD*.

Moreover, various polyamide transporter genes (HFX_0019, HFX_ 1687, HFX_6077, HFX_6079, and HFX_6080) homologous to bacterial species such as in *S. coelicolor* SCO2780, SCO3453 SCO5668, SCO5669, and SC05670 [22] and in halophilic archaea *Hfx. volcanii potA1* (HVO_A0293), *potA2* (HVO_A0294), *potB* (HVO999), and *potD* (HVO_A0300) [66] are present in the *Hfx. mediterranei* genome.

Although the presence of intracellular polyamides in *Hfx*. *mediterranei* has not yet been determined, the results described show the presence of enzymes capable of catalyzing the first step of the γ-glutamylation pathway in *Hfx*. *mediterranei.* For the first time, this provides evidence of the presence of these enzymes in *Archaea*.

## 5. Conclusions

This work successfully demonstrated that the GlnA-2 protein, in contrast to the GlnA-1 protein, shows glutamate–putrescine ligase activity. Although these halophilic proteins share structural similarities, the substitutions in essential residues for glutamine biosynthesis, which are conserved in the three domains of life, could be the reason why GlnA-2 does not present GS activity. Similarly, the *Hfx. mediterranei* growth in the presence of putrescine represents the first attempt to reveal a novel polyamine utilization pathway in the *Archaea* domain, which remains complex and unexplored. This contribution is a milestone in understanding the ecology of *Hfx. mediterranei* and the use of alternative nitrogen sources, which allows this microorganism to be competitive in its habitat and to survive under stress conditions.

## Figures and Tables

**Table 1:** Culture media used to grow *Hfx*. *mediterranei*.

Description	Culture Media	Assay
Complex medium (Hm-CM)	0.5% (*w*/*v*) yeast extract as the N and C sources.	β-galactosidase assay Gene and protein expression analysis Activity assays HM26-∆*glnA-2* characterization
Defined media (Hm-DM) with 40 mM ammonium or nitrate	Hm-DM with 40 mM NH_4_Cl or KNO_3_ as the N source and 0.5% (*w*/*v*) glucose as the C source.	β-galactosidase assay Gene and protein expression analysis HM26-∆*glnA-2* characterization
Nitrogen starvation defined media (Hm-NS)	Hm-NS without N source and 0.5% (*w*/*v*) glucose as the C source.	β-galactosidase assay Gene and protein expression analysis HM26-∆*glnA-2* characterization
Defined media (Hm-DM) with 40 mM putrescine	Hm-DM with 40 mM NH_2_(CH_2_)_4_NH_2_ as the N source and 0.5% (*w*/*v*) glucose as the C source.	β-galactosidase assay Protein expression analysis Activity assays HM26-∆*glnA-2* characterization
Complex medium (Hm-CM) with 40 mM putrescine	Hm-CM with NH_2_(CH_2_)_4_NH_2_ as the N and C sources.	Protein expression analysis Activity assays HM26-∆*glnA-2* characterization

**Table 2:** Amino acids related to glutamine synthesis of the different GlnA proteins. The substitutions of the residues are highlighted in grey.

*S. typhimurium*	*Hfx. mediterranei*	
GlnA	GlnA-1	GlnA-2
D_50_	D_63_	S_51_
S_53_	S_66_	V_53_
D_64_	D_77_	E_74_
E_129_	E_143_	E_135_
E_131_	E_145_	E_137_
Y_179_	Y_169_	A_157_
E_212_	E_200_	E_190_
E_220_	E_207_	E_197_
N_264_	N_251_	G_240_
G_265_	G_252_	T_241_
H_269_	H_256_	H_245_
H_271_	H_258_	N_247_
R_321_	R_309_	R_295_
E_327_	E_315_	G_302_
R_339_	R_327_	R_314_
R_344_	R_332_	R_319_
E_357_	E_345_	E_335_
R_359_	R_347_	R_337_
Y_397_	Y_385_	C_375_

**Table 3:** Maximum β-galactosidase activity values obtained from cultures of the *Hfx. mediterranei* transformants with pVA513 constructions in the presence of different nitrogen sources.

Media	β-galactosidase Activity in Protein Extracts of *Hfx. mediterranei* Transformants with pVA513-p-*glnA* Constructions (U/mg)	
	pVA513-p-*glnA-1*	pVA513-p-*glnA-2*
Hm-CM	1.8 ± 0.2 *	3.2 ± 0.1 *
Hm-DM with 40 mM ammonium	3.5 ± 0.5 *	3.2 ± 0.2 *
Hm-DM with 40 mM nitrate	5.6 ± 0.5 *	3.1 ± 0.3 *
Hm-DM with 40 mM putrescine	3.2 ± 0.6 *	3.7 ± 0.4 *
Hm-NS	6.17 ± 0.06 **	2.8 ± 0.2 **
	5.5 ± 0.8 ***	2.9 ± 0.2 ***

* In the middle of exponential phase. ** At 24 h of nitrogen deficiency. *** At 72 h of nitrogen deficiency.

## Data Availability

The data presented in this study are available within the article.

## Supplementary Materials

The following are available online at https://www.mdpi.com/article/10.3390/biom11081156/s1. Table S1: Primers sequences used in the promoter region cloning and in the analysis of expression at the transcriptional level by RT-PCR. Figure S1: Putative promoter regions of *glnA-1* and *glnA-2*. (a) Promoter region of *glnA-1*. (b) Promoter region of *glnA-2*. The start codon (ATG) is underlined in red and the possible transcription start site is shaded in blue. The sequences corresponding to the TATA box and the BRE region are outlined in purple and green, respectively. Figure S2: Cell growth and β-galactosidase activity of *Hfx. mediterranei* transformants pVA513-p-*glnA-1* in different culture media. (a) Hm-CM. (b) Hm-DM with 40 mM ammonium as the nitrogen source. (c) Hm-DM with 40 mM nitrate as the nitrogen source. (d) Hm-NS. Figure S3: Cell growth and β-galactosidase activity of *Hfx. mediterranei* transformants pVA513-p-*glnA-2* in different culture media. (a) Hm-CM. (b) Hm-DM with 40 mM ammonium as the nitrogen source. (c) Hm-DM with 40 mM nitrate as the nitrogen source. (d) Hm-NS. Figure S4: Cell growth and β-galactosidase activity of *Hfx. mediterranei* transformants pVA513-p-*glnA* in Hm-DM with 40 mM putrescine. (a) *Hfx. mediterranei* transformants pVA513-p-*glnA-1*. (b) *Hfx. mediterranei* transformants pVA513-p-*glnA-2*. Figure S5: Growth curves of the *Hfx. mediterranei* R4 in the presence of putrescine. All conditions are represented in different colors: ● Hm-DM with putrescine as the sole nitrogen source (with 0.5% glucose). ● Hm-DM with putrescine as the sole carbon source (without glucose, with ammonia as nitrogen source). ● Hm-CM with putrescine. Figure S6: Determination of the molecular mass of the recombinant protein GlnA-2 by chromatography on Sephacryl S-300. The standard proteins are represented in gray whereas the GlnA-2 protein is represented in blue. Figure S7: Generation and confirmation of *glnA* gene deletion mutants. (a). Genomic organization of wild type (HM26) and pop-out mutants (HM26-Δ*glnA*-*2*). Restriction sites of *Bbrp*I are represented as vertical circles (blue in the HM26, or purple in the mutant HM26-Δ*glnA-2*). The sizes of the fragments estimated for the identification of each version of the gene by Southern blot analysis are shown in black. (b). Southern blot of *glnA-2* pop-out mutants. Lane 1: DNA marker molecular weight Marker III, DIG_labeled (Roche). Lane 2-11: Mutant emergence. Lane 12: HM26. Lane 13: DNA marker molecular weight Marker III, DIG_labeled (Roche). Figure S8: Characterization of the HM26-∆*glnA-2* mutants depending on nitrogen source. (a) Hm-CM in the presence or absence of putrescine. (b) Hm-DM with different ammonium concentrations. (c) Hm-DM with different nitrate concentrations. (d) Hm-DM with different putrescine concentrations. The *p*-value is represented with asterisks corresponding to * (*p*-value< 0.05); ** (*p*-value < 0.01); *** (*p*-value < 0.001).
