# Supplementary material for: Novel Glutamate–Putrescine Ligase Activity in Haloferax mediterranei: A New Function for glnA-2 Gene
Source: Biomolecules. 2021 Aug 4;11(8):1156. doi: 10.3390/biom11081156 (PMC8394153; doi:10.3390/biom11081156)
Supplement: Supplementary file 1 [file biomolecules-11-01156-s001.zip › biomolecules-1301820-supplementary.pdf]

Supplementary material

Table S1. Primers sequences used in the promoter region cloning and in the analysis of expression at the transcriptional level by RT-PCR.

Primer	*Sequence 5´→3´*	*Tm (ºC)*	*Process*
p-glnA-1F	CGTAACTATTCGAAGCGTAGCTC	55	Promoter region cloning
p-glnA-1R	CGGTGAGTACCATGGCTTCCGTCTTT	57	
p-glnA-2F	CGTATTTCATATAAGCTTTACGTGTCGGTG	57	
p-glnA-2R	GGATTTGACCATGGATGTGTCGCTCATTAG	58	
glnA-1expF	GAATCATATGACGGAAGACAACGTA	54	RT-PCR
glnA-1expR	GCGCACGAAACCGTCGATA	55	
glnA-2expF	GCGAGGAACATATGAGCGACACA	57	
glnA-2expR	CATCGAATCGACTATTCCTGTTTCG	56	

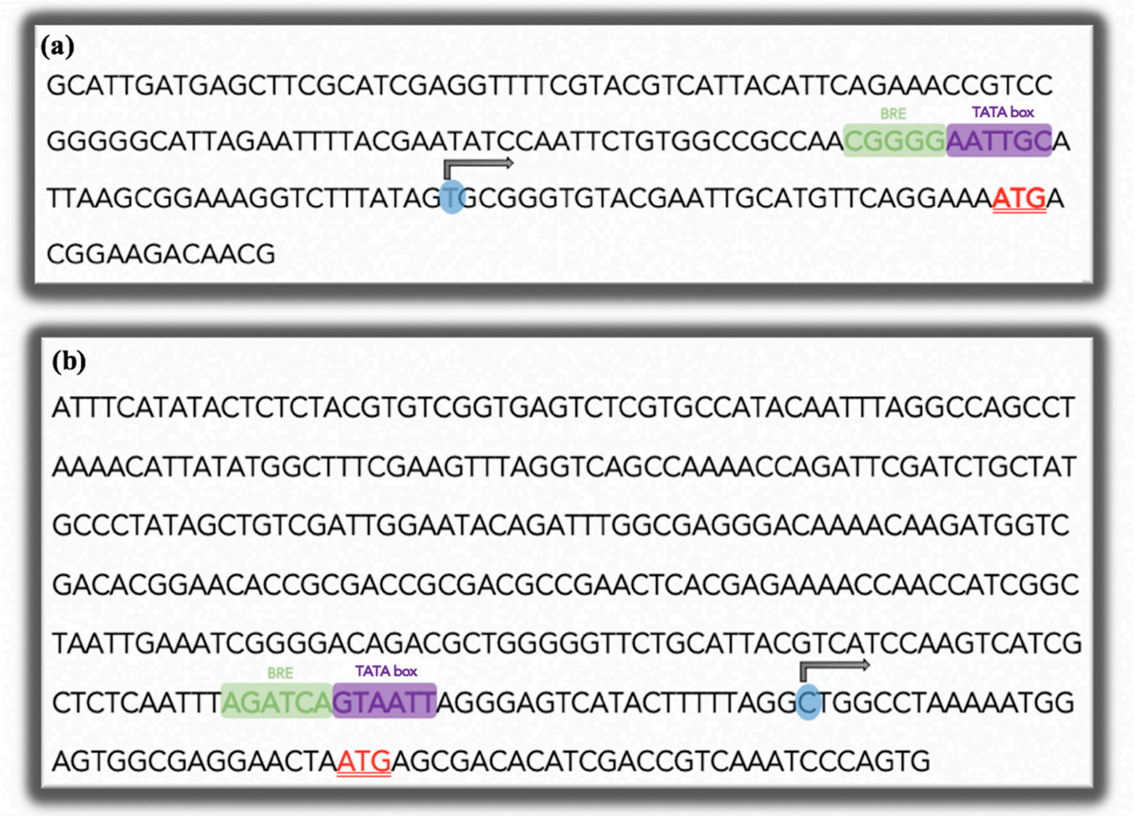

Figure S1. Putative promoter regions of *glnA-1* and *glnA-2*. (a) Promoter region of *glnA-1*. **(b)** Promoter region of *glnA-2*. The start codon (ATG) is underlined in red and the possible transcription start site is shaded in blue. The sequences corresponding to the TATA box and the BRE region are outlined in purple and green respectively.

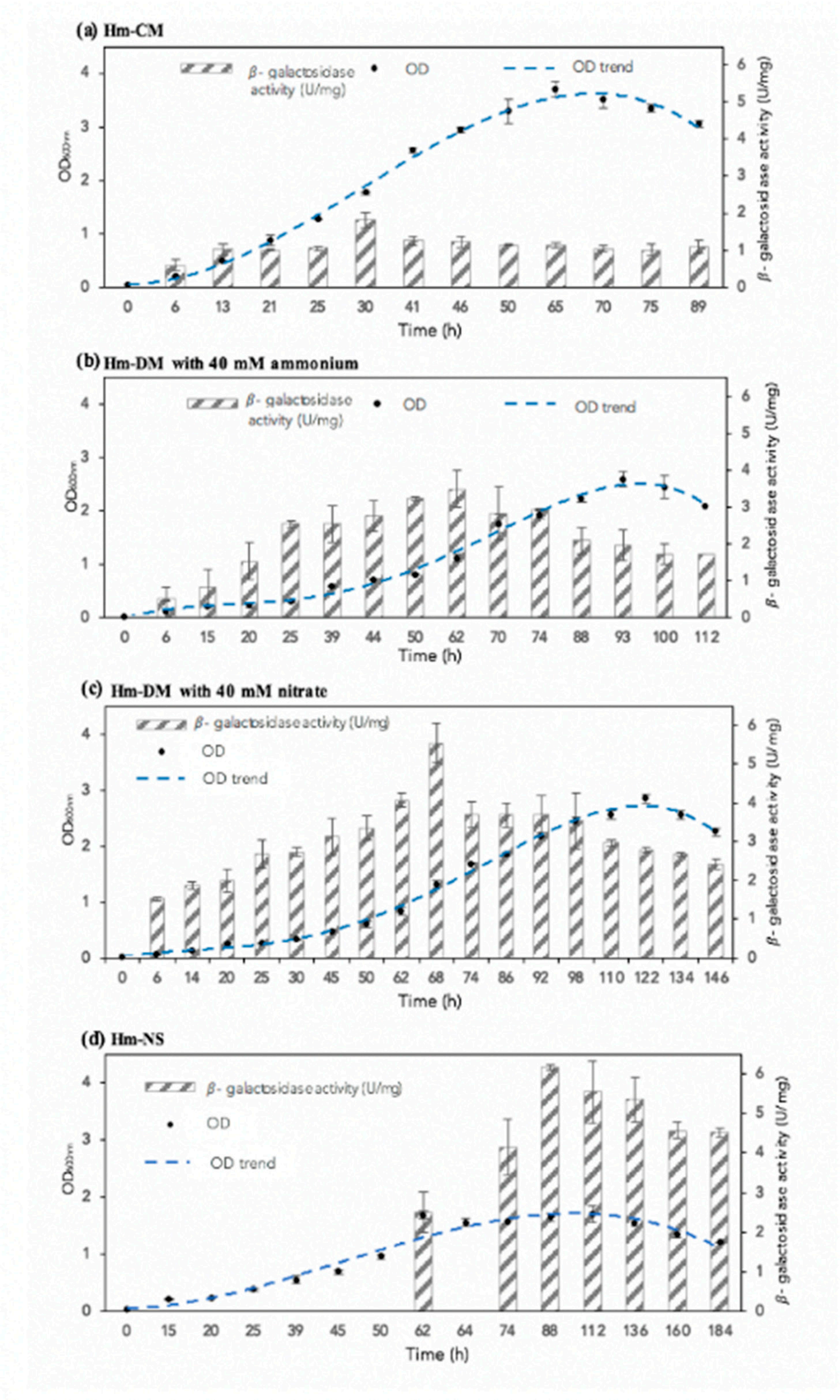

Figure S2. Cell growth and β-galactosidase activity of *Hfx. mediterranei* transformants pVA513-*p-glnA-1* in different culture media. (a) Hm-CM. **(b)** Hm-DM with 40 mM ammonium as nitrogen source. **(c)** Hm-DM with 40 mM nitrate as nitrogen source. **(d)** Hm-NS.

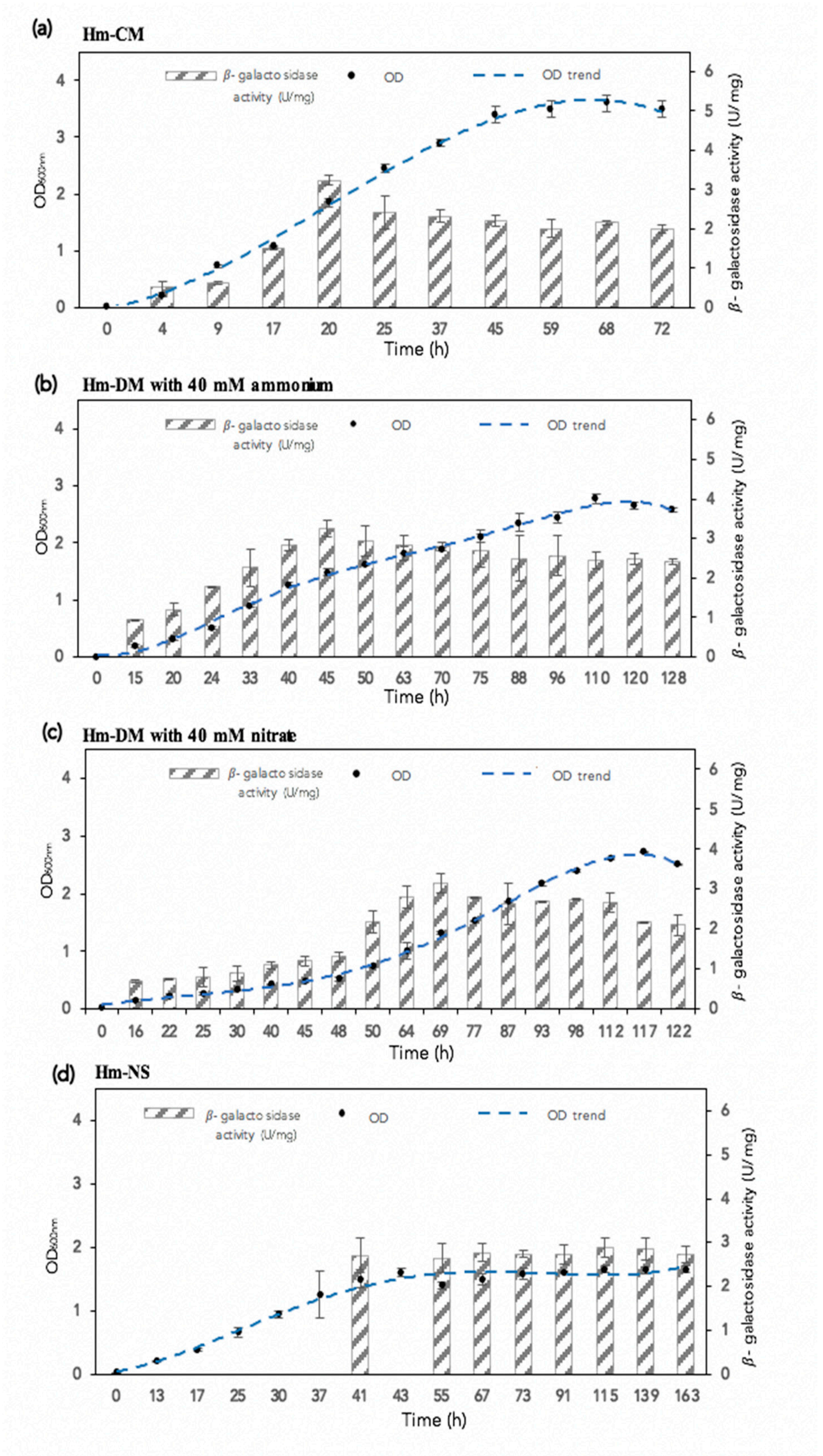

Figure S3. Cell growth and β-galactosidase activity of *Hfx. mediterranei* transformants pVA513-*p-glnA*-2 in different culture media. (a) Hm-CM. **(b)** Hm-DM with 40 mM ammonium as nitrogen source. **(c)** Hm-DM with 40 mM nitrate as nitrogen source. **(d)** Hm-NS.

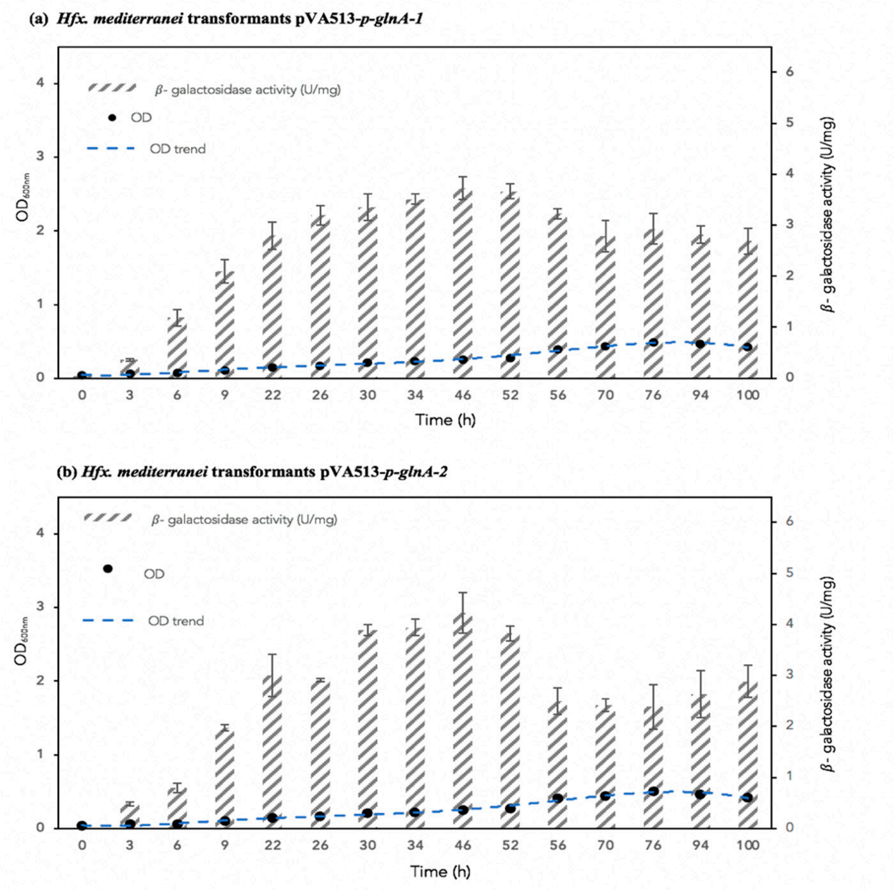

Figure S4. Cell growth and β-galactosidase activity of *Hfx. mediterranei* transformants pVA513-*p-glnA* in Hm-DM with 40 mM putrescine. (a) *Hfx. mediterranei* transformants pVA513-*p-glnA-1*. **(b)** *Hfx. mediterranei* transformants pVA513-*p-glnA-2*.

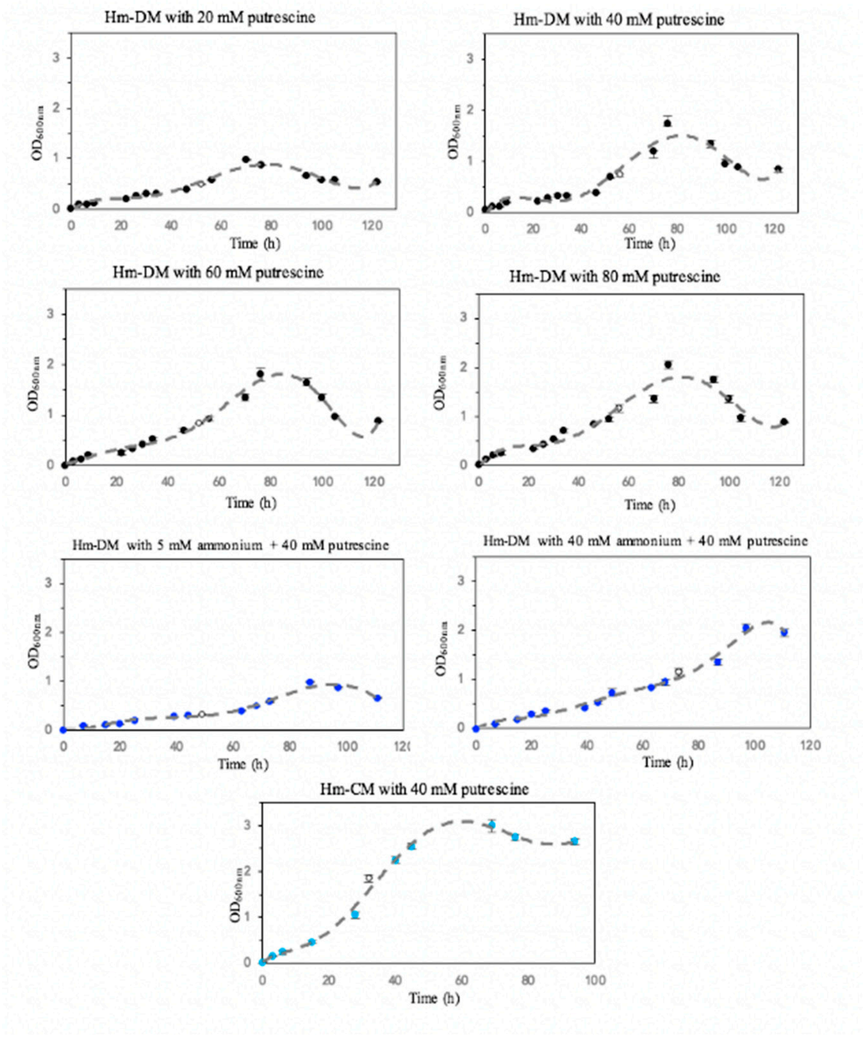

Figure S5. Growth curves of the *Hfx. mediterranei* R4 in the presence of putrescine. All conditions are represented in different colors: ● Hm-DM with putrescine as the sole nitrogen source (with 0.5% glucose). ●Hm-DM with putrescine as the sole carbon source (without glucose, with ammonia as nitrogen source). ● Hm-CM with putrescine.

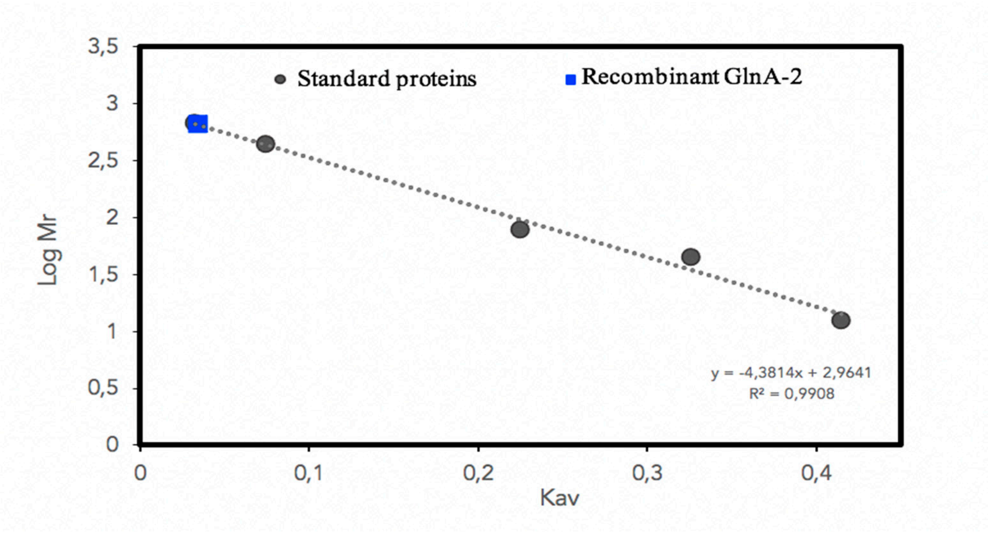

Figure S6. Determination of the molecular mass of the recombinant protein GlnA-2 by chromatography on Sephacryl S-300. The standard proteins are represented in grey whereas the GlnA-2 protein is represented in blue.

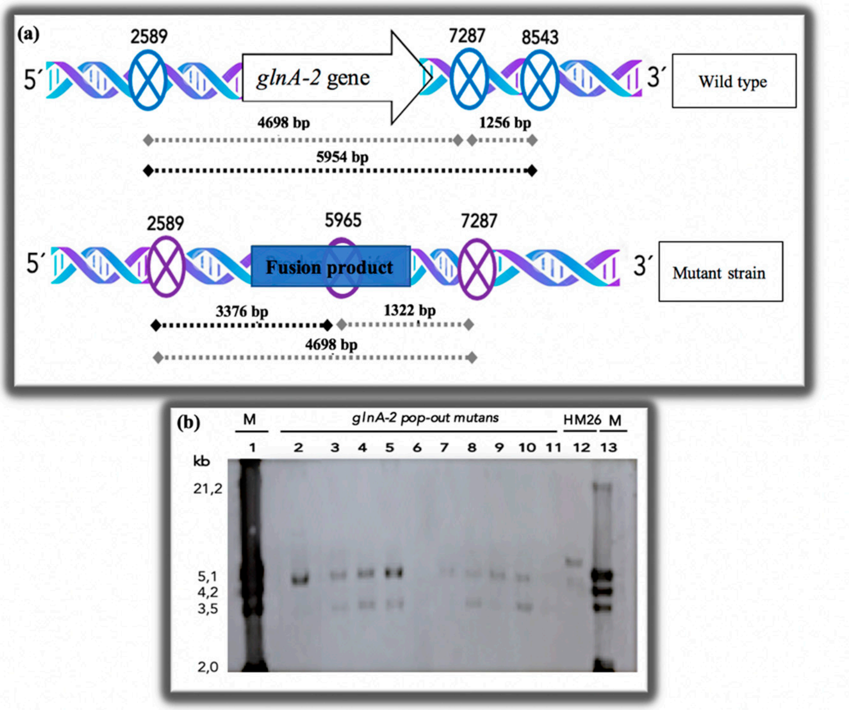

Figure S7. Generation and confirmation of *glnA* gene deletion mutants. (a) Genomic organization of wild type (HM26) and pop-out mutants (HM26-Δ*glnA-2*). Restriction sites of *Bbrp*I are represented as vertical circles (blue in the HM26 or purple in the mutant HM26-Δ*glnA-2*). The sizes of the fragments estimated for the identification of each version of the gene by Southern blot analysis are shown in black. **(b)** Southern blot of *glnA-2* pop-out mutants. Lane 1: DNA molecular weight Marker III, DIG labeled (Roche). Lane 2-11: Deletion mutants screening. Lane 12: HM26. Lane 13: DNA molecular weight Marker III, DIG labeled (Roche).

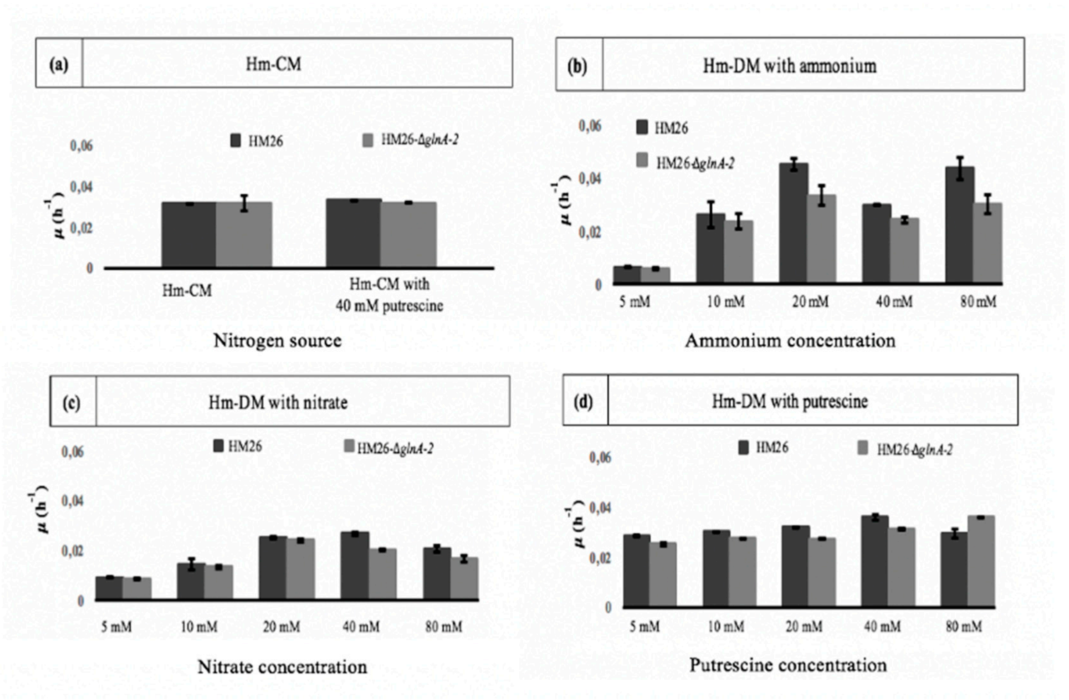

Figure S8. Characterization of the HM26-Δ*glnA*-2 mutants depending on nitrogen source. (a) Hm-CM in the presence or absence of putrescine. **(b)** Hm-DM with different ammonium concentrations. **(c)** Hm-DM with different nitrate concentrations. **(d)** Hm-DM with different putrescine concentrations. The *p*-value is represented with asterisks corresponding to * (*p*-value < 0.05); ** (*p*-value < 0.01); *** (*p*-value < 0.001).
